# Successful functional outcomes and return to sport rate can be achieved after surgery for acute Achilles tendon rupture: A systematic review

**DOI:** 10.1002/jeo2.70469

**Published:** 2025-10-28

**Authors:** Erminia Cofano, Stefano Colace, Federico Piro, Umile Giuseppe Longo, Pieter D'Hooghe, John G. Kennedy, Alberto Marangon, SIAGASCOT, Giorgio Gasparini, Michele Mercurio

**Affiliations:** ^1^ Department of Orthopaedic and Trauma Surgery, “Magna Graecia” University “Mater Domini” University Hospital Catanzaro Italy; ^2^ Department of Medicine and Surgery Fondazione Policlinico Universitario Campus Bio‐Medico Roma Italy; ^3^ Research Unit of Orthopaedic and Trauma Surgery, Department of Medicine and Surgery Università Campus Bio‐Medico di Roma Rome Italy; ^4^ Department of Orthopedic Surgery Aspetar Orthopaedic and Sports Medicine Hospital Doha Qatar; ^5^ Division of Foot and Ankle Surgery NYU Langone Health New York; ^6^ Clinic San Francesco Verona Italy; ^7^ Research Center on Musculoskeletal Health, MusculoSkeletal Health@UMG Magna Graecia University Catanzaro Italy

**Keywords:** Achilles tendon rupture, complications, functional outcomes, return to sport

## Abstract

**Purpose:**

Achilles tendon disorders are frequently seen in sports, and its rupture is one of the most common and debilitating injury. Among the most used surgical techniques there are traditional open surgeries, minimally invasive, and percutaneous techniques. The choice of technique often depends on the nature of the injury, the athlete's profile, and the surgeon's preferences. This systematic review aimed to analyze the functional outcomes, return to sport (RTS) rate and time, and complications in patients who underwent surgical repair for acute Achilles tendon lesions.

**Methods:**

The PubMed, MEDLINE, Scopus, and Cochrane Central databases were used for the research, and 9 studies were included. The first author, journal name, year of publication, patient demographics, type of sports, level of play, dominant limb and follow‐up period were recorded for each article. Data extracted for quantitative analysis included different types of lesions, types of surgical repair, RTS rate and time, the visual analog scale (VAS) for pain, the AOFAS score, the Tegner score, and the ATRS score, and the number and types of complications.

**Results:**

A total of 748 patients who underwent surgical repair of Achilles tendon were identified. Male patients represented 84% of the cases. The frequency‐weighted mean age at the time of the operation was 40.7 ± 11.8 years, and the frequency‐weighted mean follow‐up was 40.9 ± 11.7 months. The postoperative functional outcomes improved. A total of 579 patients (77.4%) returned to sport. Postoperative infection was reported in 25 patients (3.3%) and Achilles tendon re‐rupture was reported in 17 patients (2.3%).

**Conclusions:**

Patients who underwent Achilles tendon surgical repair reported successful functional outcomes and low postoperative pain scores after a mean 3.5‐year follow‐up. Postoperative AOFAS and Tegner scores higher than the normative values can be achieved. The RTS rate was 77% with a mean time of 8.1 months. Postoperative infection and tendon re‐rupture were the most common reported complications.

**Level of Evidence:**

Level III.

**Abbreviations**
RTS: return to sport
VAS: visual analog scale

## INTRODUCTION

Achilles tendon plays a crucial role in walking, running and explosive movements typical of many sports disciplines. It's formed from the combination of the distal tendinous portions of the gastrocnemius and soleus muscles and inserts into the distal‐posterior aspect of the calcaneus [37]. Its rupture is one of the most common and debilitating sports injury with an incidence of 31 per 100,000 per year, especially from 37‐ to 44‐year active population, with a higher incidence in males [6, 8]. Achilles tendon rupture leads an interruption of physical activity and a significant psychological impact for the patient [30].

Achilles tendon disorders are frequently seen in sports including running and jumping, such as basketball, soccer and tennis [7]. Risk factors include intense physical activity without adequate warm‐up, inappropriate footwear, and pre‐existing conditions such as chronic tendinopathy, inflammatory issues, autoimmune disorders, collagen irregularities, infections, exposure to fluoroquinolone antibiotics, the use of systemic or injectable steroids and repetitive microtrauma [10, 34].

Among the most used surgical techniques, there are traditional open surgeries, which offer wide exposure of the tendon but can require longer recovery times, minimally invasive techniques, which reduce the risk of skin complications, and percutaneous techniques, which further minimize invasiveness but may carry a higher risk of suboptimal repair. The choice of technique often depends on the nature of the injury, the athlete's profile, but also, significantly, on the surgeon's preferences. For this reason, there is currently no real consensus in the literature [2, 13]. Considering the type of patients, typically active and sporty, returning to sport is one of the aims of surgery. Return to sport (RTS) depends on several factors, including the patient's age, type of injury, surgical technique used, and rehabilitation protocol used during the postoperative period. The goal is to allow the patient to return to their preinjury competition level timely, safely, and effectively [19].

The aim of this systematic review was to analyze the functional outcomes, RTS rate and time, and complications in patients who underwent surgical repair for acute Achilles tendon lesions.

## MATERIALS AND METHODS

### Search strategy

A systematic review of the published literature was conducted and reported according to the Preferred Reporting Items for Systematic Reviews and Meta‐Analyses (PRISMA) statement. The study protocol was registered in PROSPERO (CRD42025649709). The PubMed, MedLine, Scopus and Cochrane Central databases were searched in October 2024. The search terms used to retrieve relevant articles were ‘Achilles’ AND ‘Tendon’, AND ‘return to’, AND ‘sport’, OR ‘play’ OR ‘performance’. Two authors (S.C. and F.P.) independently screened the titles and abstracts to identify articles for inclusion, contacting a third senior author (MM) in cases of major discrepancies. We searched for additional articles by examining the reference lists of every article included and the gray literature available at our institution. The articles were selected based on the following PICO model: (1) Population: patients with acute Achilles tendon rupture; (2) Intervention: patients who underwent surgical repair of Achilles tendon; (3) Comparator: all studies were included irrespective of the presence or absence of comparator or control groups; (4) Outcome: patients assessed for functional outcomes, complications and RTS rate and time.

### Inclusion criteria and study selection

The inclusion criteria were applied during the title, abstract, and full‐text screenings; these were defined as follows: (1) observational studies including case‐control, cohort studies, and randomized controlled trials (RCTs); (2) reporting outcomes, complications and RTS rate or time of surgical repair of acute Achilles tendon rupture; (3) reporting of >10 surgically treated cases; (4) a minimum mean follow‐up of >12 months and (5) articles written in English. Other reviews, case reports, articles without outcomes or results, articles in which a conservative treatment has been applied, cadaveric or biomechanical studies, technical notes, editorials, letters to the editor, and expert opinions were excluded from the analysis but considered for the discussion.

### Data extraction and quality assessment

Two authors (S.C. and F.P.) performed comprehensive data extraction from the included articles. The first author, journal name, year of publication, patient demographics, type of sports, level of play, dominant limb, and follow‐up period were recorded for each article. Data extracted for quantitative analysis included different types of lesions, type of surgical repair, RTS rate and time, patient‐reported outcome measures (PROMs), that were, the visual analog scale (VAS) for pain, the AOFAS score, the Tegner score and the ATRS score, as well as the number and types of complications.

A methodological quality assessment was conducted independently by three authors (M.M., S.C., and F.P.); cohort studies were assessed using the Modified Newcastle–Ottawava Quality Assessment Scale. The discrepancies were resolved by consulting a senior reviewer with over 30 years of experience in tendon surgery (GG). Details of the quality assessment are shown in Table 1.

**Table 1:** Quality assessment of included studies according to the Modified Newcastle–Ottawa scale.

Study author (year)	Criteria								Total	Quality
	1	2	3	4	5	6	7	8		
Saxena A [27]	1	1	1	1	2	1	1	1	9	High
Caruso G [4]	1	1	1	1	2	1	1	1	9	High
Jallageas R [12]	1	0	1	1	2	1	1	1	8	High
Biz C [3]	1	1	1	1	2	1	1	1	9	High
Matsumae Y [18]	1	0	1	1	2	1	1	1	8	High
Sarman H [26]	1	1	1	1	2	1	1	1	9	High
Yang S [39]	1	1	1	1	2	1	1	1	9	High
Abdelatif NMN [1]	1	0	1	1	2	1	1	1	8	High
Abdelatif NMN [1]	1	0	1	1	2	1	1	1	8	High

*Note*: Based on the total score, quality was classified as ‘low’ (0–3), ‘moderate’ (4–6) and ‘high’ (7–9). Criterion number (in bold): 1, representativeness of the exposed cohort; 2, selection of the nonexposed cohort; 3, ascertainment of exposure; 4, demonstration that outcome of interest was not present at start of study; 5, comparability of cohorts on the basis of the design or analysis; 6, assessment of outcome; 7, was follow‐up long enough for outcomes to occur?; 8, adequacy of follow up of cohorts. Each study was awarded a maximum of one or two points for each numbered item within categories, based on the Modified Newcastle‐Ottawa scale rules.

A substantial interobserver agreement (Cohen's kappa coefficients ranging between 0.61 and 0.73) was reported.

### Data synthesis

The quantitative data were organized for statistical analysis; all data were collected, measured, and reported with one‐decimal accuracy. Weighted means and standard deviations (SD) were calculated for data concerning demographic characteristics and outcomes. When SDs were not directly provided, they were calculated with the equation [max range– min range/4] to allow for statistical aggregation [20]. The normative values of the AOFAS and the Tegner scores were considered [17, 29]. The weighted mean and SD comparisons between postoperative and normative scores were performed using unpaired *t*‐tests; the comparisons have been performed only when the data were reported in at least two studies. All tests were performed with IBM SPSS Statistics software (version 25.0; IBM Corp.) and GraphPad Prism (version 7.0; GraphPad Software Inc.). Confidence intervals (CIs) were set at 95%, and a *p *< 0.05 was considered significant.

## RESULTS

**Figure 1:**
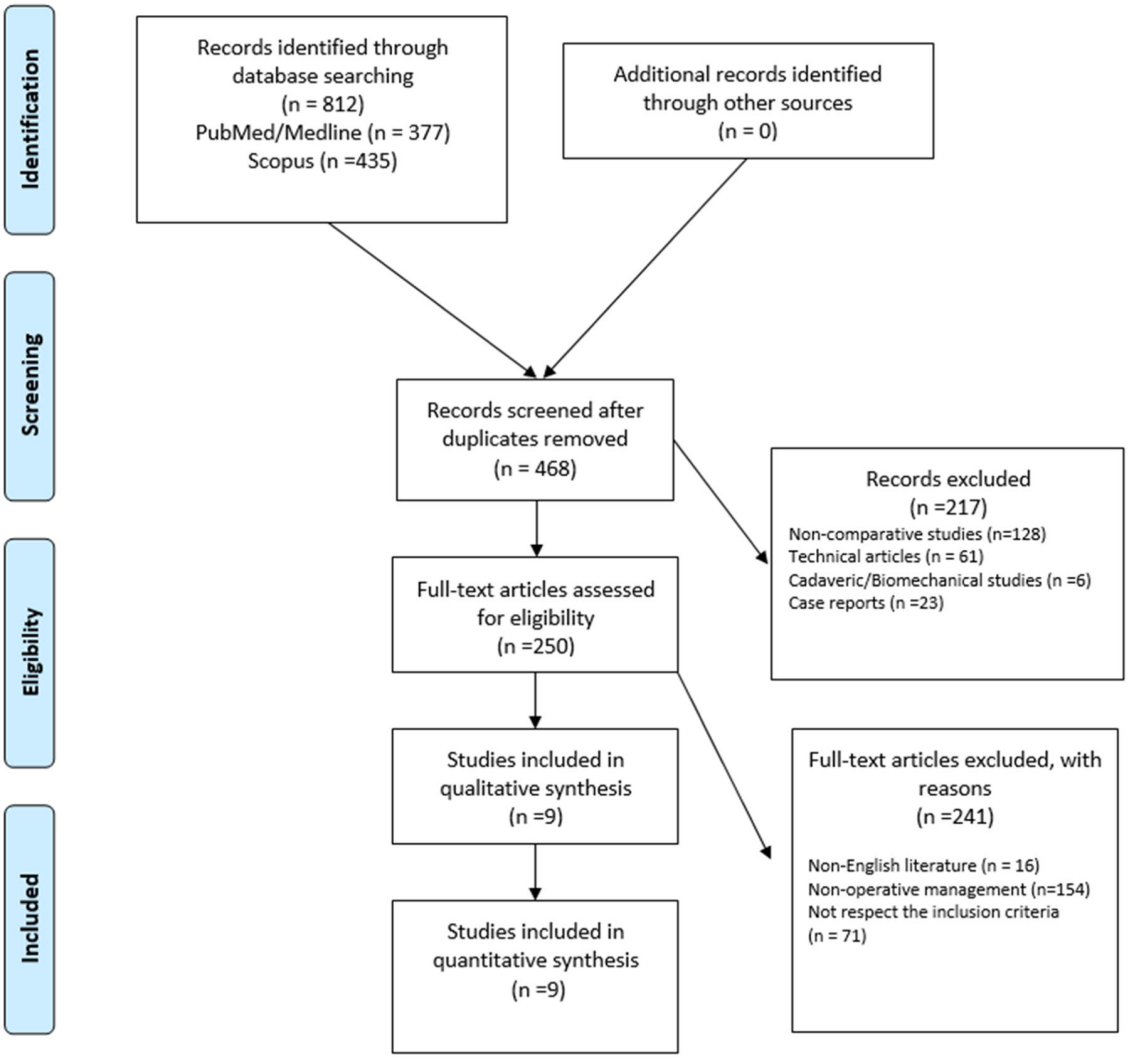
Preferred Reporting Items for Systematic Review and Meta‐Analysis (PRISMA) flowchart for the searching and identification of included studies.

A total of 812 relevant articles were identified through the initial search, 468 abstracts were screened, and 250 full‐text articles were assessed for eligibility based on our inclusion criteria, resulting in 9 studies that were eligible for the systematic review (Figure 1).

A total of 748 patients who underwent surgical repair of Achilles tendon were identified. The baseline characteristics of the patients are summarized in Table 2.

**Table 2:** Characteristics of included studies.

Author	Journal	Year of publication	Level of evidence	Years of study	Patients demographics												
					Number of patients (*N*)	Sex (*N*)		Age (years)			FU (months)			Level of play		Type of surgical technique	
						M	F	Mean	SD	Range	Mean	SD	Range	Professional	Nonprofessional	Open repair	Minimal invasive repair
Abdelatif NMN et al.	Foot & ankle international	2022	II	2015–2018	27	27	0	31	3.8	NA	46.2	10.9	NA	27	0	27	0
Abdelatif NMN et al.	Foot & ankle international	2022	III	2016–2018	117	110	7	36.2	8.2	NA	42.8	9	30–64	45	72	58	59
Biz C et al.	Medicina	2021	NA	2010–2014	90	74	16	40.9	7.6	18–50	116.4	28.8	NA	0	90	0	90
Caruso G et al.	European Journal of Orthopaedic Surgery & Traumatology	2024	NA	2014–2021	155	143	12	48.3	14.7	NA	72.3	31.6	NA	0	155	53	103
Jallageas et al.	Orthopaedic & Traumatology: Surgery & Research	2013	IV	2005–2009	31	26	5	38.2	12.1	18–71	15	NA	NA	16	15	15	16
Matsumae Y et al.	Journal of clinical medicine	2024	NA	2017–2022	27	21	6	35.8	10.3	NA	32.4	9.1	NA	27	0	27	0
Sarman H et al.	The Journal of Foot & ankle Surgery	2016	III	1998–2011	45	36	9	39.5	7.25	27–56	43.7	27.5	6–116	0	45	21	24
Saxena et al.	The Journal of Foot & ankle Surgery	2021	III	2001–2011	188	149	39	42.1	12	NA	85.4	56.3	NA	0	188	85	103
Yang S et al.	BMC musculoskeletal disroders	2023	III	NA	68	42	26	35.1	6.9	18–50	52	16.3	NA	0	68	68	0

Abbreviations: FU, follow‐up; NA, not available; SD, standard deviation.

Overall, male patients represented 84% of the cases. The frequency‐weighted mean age at the time of the operation was 40.7 ± 11.8 years, and the frequency‐weighted mean follow‐up was 40.9 ± 11.7 months. The prevalence of smoking habits was 4.3%. The most common sports practiced by the patients were soccer (15%), running (6%), tennis (1.9%), basketball (1.5%), and handball (1.3%). Considering the level of play, 15.4% of the patients played sport at a professional level while 633 (84.6%) were nonprofessional athletes. The injured limb was the right in 315 (42%) patients while in 311 (41.6%) patients was the left one.

All the patients had an acute Achilles tendon lesion. The lesion was in the proximal part in 13 patients (1.8%), in the midportion in 729 (97.4%) patients, and in the distal part in 6 patients (0.8%). The overall weighted meantime between the injury and surgery was 14 days. A total of 47.2% (354) of the cases underwent open surgery while 52.8% (395) underwent a minimally invasive repair. Tenorrhaphy was performed in 637 patients (85.2%). In 111 patients (14.8%) the lesion was repaired with a reconstruction with a graft: in 85 patients (76.6%) a flexor hallucis longus was used.

### Functional outcomes and RTS

The postoperative VAS score was measured in 4 studies [3, 12, 27, 39], involving 377 cases, with a mean value of 2.5 ± 1.9.

The postoperative AOFAS score was evaluated in 6 studies [1, 3, 12, 18, 26, 39], involving 378 patients, with a mean value of 95.2 ± 7.3 that was higher than the normative AOFAS age‐related data (40–49 years, 91.1 ± 2.3) (*p* < 0.001). The mean postoperative AOFAS score was 105% that of healthy individuals.

In 2 studies [1, 39] the postoperative Tegner score was evaluated in 185 patients with a mean value of 6.0 ± 2.2 that was higher than the normative Tegner data (4 ± 2.1) (*p* < 0.001). The mean postoperative Tegner score was 150% that of healthy individuals.

The postoperative ATRS score was evaluated in 4 studies [1, 3, 4, 39], involving 430 patients, with a mean value of 94 ± 7.2.

A total of 579 patients (77.4%) returned to sport. The overall weighted mean time between surgery and RTS was 8.1 ± 4.5 months.

The postoperative functional outcomes are shown in Table 3.

**Table 3:** Postoperative functional outcomes.

Postoperative functional outcomes	Mean	SD
VAS	2.5	1.9
AOFAS score	95.2	7.3
TEGNER score	6	2
ATRS	94	7.2
Return to sport (time–months)	8.1	4.5
Return to sport (rate−%)	77.4%	

Abbreviations: SD, standard deviation; VAS, visual analogue scale.

### Complications

A postoperative infection was reported in 25 patients (3.3%). The Achilles tendon re‐rupture was reported in 17 patients (2.3%). A suture reaction occurred in 13 patients (1.7%). The venous thromboembolism and transient sural nerve palsy were both reported in 9 patients (1.2% for each).

## DISCUSSION

The most important finding of this study was that patients undergoing surgical repair for an acute Achilles tendon lesion of the midportion report, on average, successful functional outcomes and low postoperative pain scores after a mean 3.5‐year follow‐up. The postoperative AOFAS and Tegner scores of patients in the included studies were higher than the normative values. The RTS rate was 77% with a mean time of 8.1 months. Postoperative infection and tendon re‐rupture were the most common reported complications.

This study updates the current evidence from previous systematic reviews on this topic the literature is limited. There has been an increased incidence of acute Achilles tendon injuries in the population, especially among athletes. In particular, in professional athletes, this type of injury could lead to the end of their professional career [23, 35, 36]. The data on how to manage this type of lesion and what to aspect after surgery are limited [11, 31].

We reported successful functional outcomes and low postoperative pain scores after a mean 3.5‐year follow‐up in patients with an acute Achilles tendon injury underwent surgery. Moreover, the postoperative AOFAS and Tegner scores were higher than the normative values. This data concurs with those reported by Liu et al. [14] that showed a notable increase of 45 points between preoperative and postoperative AOFAS scores with a mean AOFAS score at the final follow‐up of 97.4 points. Also, Abdelatif et al. [1] reported mean postoperative AOFAS and Tegner scores of 97.1 ± 4.2 and 6.6 ± 2.4, respectively. In this light, it should be considered that most patients included in the current review were actively sporting male with a mean age of 41 years; part of the explanation for the particularly positive functional outcomes reported can be found in the population that was examined [5].

One important goal after surgical repair of Achilles tendon rupture is to quickly RTS, and it is considered a continuum: return to participation, RTS, and return to performance [21, 22]. The level of ankle function achieved may not be an objective measure for assessing the likelihood of more active patients who want to RTS and perform at a higher physical level [22]. We next reported that the RTS rate was 77% with a mean time of 8.1 months. A metanalysis by Zellers et al. [40] noted that the RTS rate was 80% with a mean time of 6 months in patients who underwent surgical repair for acute tendon rupture. A study by Grassi et al. [11] reported a mean time of RTS of 7 months while a mean time of 9 months to return to competitive matches in soccer players with an overall rate of 96%. Maffulli et al. [16] found that the RTS in professional soccer players was 5 months. In other two studies that examined American professional football players the RTS rate were 68% and 69.4%, respectively [25, 35]. Our results were similar to those reported in the literature, but we failed to distinguish between RTS and return to performance since the available data didn't report this condition. The type of surgical approach represents an important and controversial aspect for the functional recovery and RTS. Open repair allows the surgeon to fully visualize the lesion and the tendon stumps but can expose patients to an increased risk of wound dehiscence, as well as superficial and deep infection [9, 38]. On the other side, percutaneous repair has potential advantages in terms of reduced operative time, complications, and improvement of functional scores and it has been reported that this technique might be superior to the open repair, especially in professional players. However, the incidence of sural nerve seems to be higher in comparison to the open approach. The minimally invasive approach presents numerous advantages such as the preservation of biological vascularization and the possibility of fully participating in sporting activity without suffering from loss of push‐off strength [33, 35]. Caruso et al. [4] found that one in two patients who underwent percutaneous surgery returned to their pre‐injury level compared to one‐third of patients who underwent open surgery. However, the data available in the current review did not allow a direct comparison between open and minimally invasive approaches in terms of functional results, complications and RTS.

In the current review, postoperative infection and tendon re‐rupture were the most common reported complications. Surgery is the best option to reduce the risk of re‐rupture compared to the conservative treatment but a higher risk of other procedure‐related complications should also be considered [15]. Stavenuiter et al. [32] found that the overall complications was 11.7% and the most reported were infections and re‐ruptures; the authors also found that the minimally invasive technique resulted in a higher complications rate (13.2%) than the open surgery (11.6%). Wound healing is one of the most critical complications in this surgery. Saxena et al. [28] found that a high rate of granulomas were associated with the type of suture material but not with the specific suture technique used. In this light, the non‐absorbable braided suture and high resistance suture were reported as risk factors.

The results of this study should be interpreted with caution because it has several limitations [24]. First, we excluded technical notes and case reports because the inclusion of articles with a higher risk of bias could disqualify the systematic review. Second, only studies published in the English language were included, which may have contributed to publication bias; furthermore, this search involved four major literature databases; so, we cannot exclude the possibility that additional articles could have been identified using other databases. Third, heterogeneity in terms of sample size, and a higher percentage of midportion tendon lesions and actively sporting male patients was found in the included cohorts; therefore, caution should be used in the interpretation of the results and their generalizability. Fourth, due to the lack of relative data or adequate statistical aggregation, it was not possible to compare outcomes and complications according to the type of surgery used. Clinicians should not ignore the fact that differences in patients' characteristics may favor one treatment option over another. There was also heterogeneity in terms of the type of post‐operative management. Finally, we found heterogeneity in the mean follow‐up time of the included studies. If a specific and longer follow‐up time is used, the results could potentially differ.

## CONCLUSIONS

This systematic review showed that patients undergoing surgical repair for an acute Achilles tendon lesion report, on average, successful functional outcomes and low postoperative pain scores after a mean 3.5‐year follow‐up. Postoperative AOFAS and Tegner scores higher than the normative values can be achieved. The RTS rate was 77% with a mean time of 8.1 months. Postoperative infection and tendon re‐rupture were the most common reported complications. The results of the current study may be of interest to patients, clinicians, and health professionals involved in the management of active patients.

## AUTHOR CONTRIBUTIONS

Erminia Cofano participated in the acquisition and interpretation of data, performed the statistical analysis, and drafted the manuscript. Stefano Colace participated in the acquisition and interpretation of data and drafted the manuscript. Federico Piro participated in the acquisition and interpretation of data and drafted the manuscript. Umile Giuseppe Longo drafted the manuscript. Pieter D'Hooghe participated in the interpretation of data and drafted the manuscript. John G. Kennedy participated in the acquisition and interpretation of data and drafted the manuscript. Alberto Marangon drafted the manuscript. Giorgio Gasparini conceived and coordinated the study and revised critically the manuscript. Michele Mercurio designed, conceived and coordinated the study and revised critically the manuscript, approved the final version of the manuscript as submitted. All authors approved the final version of the manuscript and agree to be accountable for all aspects of the work in ensuring that questions related to the accuracy or integrity of any part of the work are appropriately investigated and resolved.

## CONFLICT OF INTEREST STATEMENT

The authors declare no conflicts of interest.

## ETHICS STATEMENT

The authors have nothing to report.

## Data Availability

The authors have nothing to report.
